# Platelets and Multi-Organ Failure in Sepsis

**DOI:** 10.3390/ijms18102200

**Published:** 2017-10-20

**Authors:** Elisabetta Greco, Enrico Lupia, Ornella Bosco, Barbara Vizio, Giuseppe Montrucchio

**Affiliations:** Department of Medical Science, University of Turin, 10126 Turin, Italy; betta.greco5@gmail.com (E.G.); enrico.lupia@unito.it (E.L.); ornella.bosco@unito.it (O.B.); barbara.vizio@unito.it (B.V.)

**Keywords:** platelet, sepsis, multi-organ failure, thrombocytopenia, septic shock, antiplatelets, ARDS, AKI, DIC

## Abstract

Platelets have received increasing attention for their role in the pathophysiology of infectious disease, inflammation, and immunity. In sepsis, a low platelet count is a well-known biomarker for disease severity and more recently authors have focused their attention on the active role of platelets in the pathogenesis of multi-organ failure. Septic shock is characterised by a dysregulated inflammatory response, which can impair the microcirculation and lead to organ injury. Being at the crossroads between the immune system, clotting cascade, and endothelial cells, platelets seem to be an appealing central mediator and possible therapeutic target in sepsis. This review focuses on the pathogenic role of platelets in septic organ dysfunction in humans and animal models.

## 1. Introduction

Sepsis is a complex syndrome characterized by a disordered immune, endocrine, and metabolic response to infection. This exaggerated response can lead to multi-organ failure (MOF), shock, and death. According to the new definition of sepsis, organ dysfunction and a dysregulated immune host response are the key factors that differentiate infection from sepsis [1]. The severity of organ dysfunction has a prognostic value, and in clinical practice is usually classified according to the Sequential [Sepsis-related] Organ Failure Assessment (SOFA) score [2]. The pathogenesis of MOF in sepsis has been widely investigated, however, efforts to translate the findings from bench to bedside in clinical trials have failed. Widely adopted resuscitation bundles focus on early antibiotic therapy, fluid resuscitation, and blood pressure targets, however few recommendations in the recent Surviving Sepsis guidelines are based on high-quality evidence [1,2]. Furthermore, sepsis treatment often focuses on macrovascular end points (e.g., mean arterial pressure and urine output) and not microvascular and metabolic dysfunction that probably play an important role in the pathogenesis.

Hematological failure is common in patients with septic shock; the correlation between thrombocytopenia and sepsis was first demonstrated over 40 years ago [3]. Thrombocytopaenia below <50,000/µL is a strong negative prognostic marker in patients with sepsis and is thought to result from platelet activation and consumption [4,5]. Different markers of platelet function have been suggested as biomarkers for sepsis and have been shown to correlate with severity [6] (Table 1).

Platelets are anucleated cells that play an established role in hemostasis and coagulation. However, hemorrhagic complications during sepsis are rare and rarely lead to death. Attention has also been focused on their role in the immune system [15]. Platelets are able to release cytokines, recruit leukocytes, interact with bacteria and the endothelium, and contribute to microthrombi formation [16]. These mechanisms are adaptive and protective in the context of a localized infection, but become dysregulated and “maladaptive” during sepsis, contributing to organ damage [17].

In this review the following questions will be considered: What are the possible mechanisms of platelet dysfunction leading to multi-organ failure during sepsis? What evidence do we have for these mechanisms? Is platelet function a potential therapeutic target in sepsis?

## 2. Mechanisms of Platelet-Mediated Organ Damage in Sepsis

### 2.1. Role of Receptors and Transcellular Cross-Talk in Platelet Function During Sepsis

Platelet interaction with immune and endothelial cells is a well-known and conserved response against infection. Activated platelets interact with other cells via two main mechanisms: (1) expression of receptors on cellular surface; and, (2) release of cytoplasmic granules that contain immunomodulatory proteins. CD40, CD154, Toll-like receptors, TREM-1 ligand, P, and E selectin are all expressed during platelet activation. α-granules containing chemokines, adhesive proteins, and clotting factors are usually stored at cytoplasmic level and on stimulation can be released to promote immunomodulation [6].

Platelets play an important role in the guidance and activation of neutrophils, supporting leukocyte rolling, adhesion, and transmigration in peripheral vessels. Leukocytes that interact with platelets express a higher number of receptors related to infection and inflammation and have a stronger bactericidal capacity. Platelet-leukocyte complexes (PLCs) can be measured in vivo and increased numbers of PLCs have been shown both in animal models of sepsis and humans. Reduced numbers of PLCs are associated with progression of MOF [18,19] and although causality is yet to be demonstrated, it may represent an indirect sign of platelet consumption in vessels.

Following is a brief description of the mechanisms involved in platelet cross-talk with other cell-types during sepsis.

TREM-1 is a well described leukocyte receptor that is expressed in response to infection. Platelets express a ligand for TREM-1, levels of which correlate with the severity of sepsis and have been studied as a potential therapeutic target [20,21].

Platelets are also involved in the formation of Neutrophil extracellular traps (NETs), a complex web-like structure of DNA with proteolytic activity built by neutrophils, with the ability to trap microorganisms and facilitate their clearance [22]. NETs mainly play a role in very small vessels, including lung capillary and hepatic sinusoids. Small cohort studies of septic patients on ICU (Intensive Care Unit) have shown that increasing levels of circulating NET biomarkers (free DNA/myeloperoxidase complexes) correlate with multi-organ dysfunction. Animal models of severe bacterial sepsis have found that intravenous treatment with DNase reduces organ damage and improves survival [23].

Platelets show complex interactions with neutrophils and the endothelium. Vasodilation, increased permeability, adhesion of immune cells, alteration in the glycocalyx and cytokine release become dysregulated during sepsis. Ince et al. have widely reviewed the important role of endothelium in sepsis [24].

P-selectin mediated adhesion is an important platelet-endothelial-leukocyte interaction in sepsis. P-selectin is contained in platelet α-granules and is expressed on membrane surface after activation [25,26]; inhibition of P-selectin has been investigated and as a potential target, but results have to be confirmed in vivo [27].

ICAM-1 expression on endothelial cells is induced by activated platelets and promotes neutrophil adhesion. An animal model of acid-induced lung injury suggests that it may be a potential therapeutic target: inhibition of ICAM-1 mediated platelet-endothelial-neutrophil interaction resulted in increased animal survival time and less hypoxia [28].

Another interesting immunomodulatory mechanism involves microparticles (MPs). MPs are small vesicles released from the cell surface of platelets, which function as storage for coagulation factors and cytokines [29]. Elevated MP levels correlate with the severity of sepsis in clinical studies. [30,31,32]. Studies investigating the effect of intravenous microparticles in rats have resulted in deranged clotting, acute respiratory distress syndrome, and a haemodynamic syndrome typical of sepsis [33,34].

Our group has investigated the involvement of thrombopoietin (TPO) in platelet-leukocyte interaction and the development of organ damage in sepsis. TPO is the growth hormone involved in thrombopoiesis. In normal physiological states it promotes platelet production through megakaryocyte stimulation and is released by platelets themselves upon activation. TPO levels are also increased during inflammatory states [35], enhancing the response of mature platelets to several agonists, increasing platelet-leukocyte adhesion via P-selectin, increasing reactive oxygen species release and inducing IL-8 production by neutrophils and monocytes [36,37,38,39]. Our group and others have shown significantly elevated levels of TPO in both murine and human sepsis [14,40,41]. In addition to its possible role as a biomarker and pathogenic mediator of sepsis, it has been shown that inhibition of TPO prevents lung, liver, and gut damage in a cecal ligation and puncture (CLP) model of sepsis [42].

In summary, there are several receptors involved in platelet cross-talk with immune and endothelial cells during sepsis with roles leading to organ damage. To date, none of these mediators have been successfully targeted in clinical practice. However, future benefits may result from further characterization of the molecular and cellular mechanisms involved in these processes and contribute to a theragnostic approach to treating sepsis and improving mortality.

### 2.2. Platelet Involment in Microvascular and Mitochondrial Dysfunction

Platelet-endothelial adhesion, platelet-leukocyte aggregates, and NETs all contribute to the formation of microthrombi in small vessels. The cells involved release cytokines and chemokines resulting in further cellular recruitment, which can become a pathological self-sustaining dysregulated process resulting in septic shock. The formation of microthrombi in the vessels triggered by the inflammatory response and the subsequent recruitment of immune cells and platelets is known as immunothrombosis [43].

Immunothrombosis contributes to microvascular dysfunction, which is a hallmark of organ damage in sepsis [44]. Capillaries, arterioles, venules, and micro-lymphatics are all part of the microvascular network. During sepsis, even when organ perfusion is preserved, patchy areas of reduced oxygen delivery and extraction and functional shunting have been shown. Alteration of microvascular function correlates with the severity of sepsis and mortality [45,46,47].

The final acceptor of oxygen at the subcellular level is the electron transport chain in mitochondria. Mitochondrial dysfunction has been widely investigated as a possible mechanism of organ damage during sepsis. This concept is supported by the absence of widespread cellular apoptosis and necrosis in patients with MOF [48], and by the rapid recovery of organ function after the resolution of sepsis. Radical oxygen species, mitochondrial “hibernation” and uncoupling can contribute to the “metabolic stunning” at the cellular and subcellular level of septic organs [17]. Skeletal muscle mitochondrial dysfunction has been shown in both animal models and septic patients [49,50,51]. Also, cultured cells and isolated mitochondria incubated with septic serum show mitochondrial dysfunction [52,53,54]. These findings support the idea that organ damage occurs at a subcellular level driven by oxidative stress and impaired mitochondrial respiration. Mitochondrial dysfunction has also been shown in platelets during sepsis; alterations in platelet mitochondrial respiration correlate with the severity of disease [55,56]. Moreover, healthy platelets incubated with septic serum results in post-trascriptional changes including tissue factor pre-mRNA splicing that could be involved in increasing pro-coagulant activity in sepsis [57]. Interesting new evidence of the possible benefit of antioxidants and radical oxygen species scavengers (e.g., high doses of intravenous vitamin C in septic shock [58]) have recently been published and appear promising [59,60]. However, further studies on reversal of mitochondrial dysfunction in sepsis are needed.

## 3. Platelet-Mediated Organ Damage

### 3.1. Platelets and Lung Damage

Acute respiratory distress syndrome (ARDS) is one the most severe complications of sepsis and is characterised by increased alveolar-capillary barrier permeability, pulmonary oedema, and severe hypoxemia. Patients with ARDS may require intubation and mechanical ventilation and current standard therapy includes lung protective ventilation to reduce lung trauma generated by high pressure in alveoli. Barotrauma is correlated with increased inflammation and worse prognosis [61].

Leukocytes and platelet recruitment, intravascular coagulation, endothelial damage, loss of surfactant, oxidative stress are all mechanisms underlying severe lung damage in sepsis. Post mortem biopsies of patients who died with ARDS have shown excess numbers of platelets and neutrophil deposition in pulmonary vessels [62].

The literature describes several roles for platelets in the pathogenesis of ARDS, in both animal and human studies; platelet depletion has been shown to correlate with reduced recruitment of neutrophils in lung interstitium [63] and increased platelet-derived thromboxane-A2 and P-selectin correlated with increased neutrophil activation, in a mouse model of ARDS [28]. This second mechanism can be reversed, as shown by inhibition of P-selectin with antibodies and in knock out mice model of barotrauma [64].

Enhanced platelet activation has also been demonstrated in bronchoalveolar lavage of patients with ARDS [65].

### 3.2. Platelets and Disseminated Intravascular Coagulation (DIC)

About 80% of all septic patients have some degree of coagulopathy. DIC (disseminated intravascular coagulation) is a condition involving uncontrolled systemic activation of the clotting cascade leading to clotting factor consumption and microvascular thrombosis. Complications include thrombotic and hemorrhagic events. No specific treatment has been identified so far and management of DIC complication can be very challenging in acute phases of sepsis. For all these reasons mortality is very high. Several anticoagulation treatments have been studied in clinical trials with no improvement in mortality [66].

Platelets play a key role in normal hemostasis, stabilising the clot at endothelial level. During inflammatory states, platelets also act as amplifiers for clotting factor activation and cell recruitment. In particular, platelet-neutrophil aggregates are platforms for thrombi generation and that is the trigger for NET release [67,68].

A selective mechanism that is able to target infection-induced uncontrolled coagulation and preserve normal coagulation, needs to be identified. NET inhibition seems to be the most appealing approach, in particular inhibition of the NET associated protein histone H4, which has been shown to protect from DIC in an animal model of sepsis [69]. Also, intravenous DNAse has shown to successfully breakdown NETs and reduce organ damage [23,70].

### 3.3. Platelets and Acute Kidney Injury (AKI)

AKI (acute kidney injury) is a frequent complication of sepsis, the pathophysiology and management of which are still controversial. Leukocyte infiltration in the septic kidney has been widely shown in animal models and septic patient; leukocyte depletion seems to reduce renal injury [71]. P-selectin stored in α-granules of platelets and in endothelial cells is involved in leukocyte recruitment in septic kidney. Blocking P-selectin protects mice from AKI by attenuating neutrophil recruitment into the kidney [72]. MPs are an interesting pathogenetic mechanism, and a correlation between MPs and blood urea nitrogen in AKI has been indicated in septic patients [30].

### 3.4. Platelets and Septic Cardiomyopathy

A certain degree of cardiac impairment has been demonstrated in up to 80% of septic patients, although agreement on a definition of septic cardiomyopathy has still not been reached. Interestingly, sepsis induced cardiac impairment resolves within 7–10 days. Humoral factors are regarded as the most likely cause, although nitric oxide modulation and leukocyte recruitment have also been suggested in the pathogenesis [73].

Incubation of platelet-derived MPs with isolated heart and papillary muscle preparations induces a decrease in myocardial contraction in vitro [74], however in vivo studies are lacking.

Our group has shown that pretreatment with TPO prevents septic serum-induced myocardial contractility depression [75].

## 4. Antiplatelets and Prevention of Organ Damage during Sepsis

Acetylsalicylic acid (ASA) and P2Y12 inhibitors are prescribed worldwide in the secondary prevention of cardiovascular disease. ASA inhibits platelet function through the blocking of COX-1. Clopidogrel is a platelet membrane P2Y12 receptor inhibitor, thereby preventing ADP (adenosine diphosphate) activation. Antiplatelet drugs have an important anti-inflammatory effect, and can reduce C-reactive protein, P-selectin, and leukocyte-platelet aggregates [76,77], and have therefore been proposed as possible targets for sepsis prevention and treatment.

Animal studies, retrospective, and observational clinical studies have shown that antiplatelet drugs may reduce MOF, hospital stay, and mortality in critically ill patients, including those affected by sepsis [78,79,80,81,82,83]. Despite initially encouraging results, a recent propensity-matched analysis in a cohort of 972 patients has not confirmed the results of retrospective analysis, showing no improvement in mortality in-patient on a pre-existing antiplatelet regimen [84].

LIPS-A trial is a recently completed randomised controlled trial (RCT) that has included 195 patients per group at risk of developing ARDS. Treatment with aspirin was not correlated with improvement in terms of risk of developing lung injury [85].

Other ongoing randomized controlled trials are expected to give a definitive answer. Details of ongoing RCTs are summarised in Table 2.

## 5. Conclusions

Emerging evidence highlights the role of platelets as immune mediators. Given their interaction with immune cells, endothelium, and clotting factors, and the widespread use of antiplatelet drugs in the general population, platelets seem an appealing therapeutic target in sepsis. Data from studies of both animal models and septic patients have shown the contribution of platelets to multi-organ dysfunction. The ongoing randomized controlled trials are expected to give more answers on the clinical effect of platelet inhibition in sepsis prevention and treatment.

## Figures and Tables

**Table 1:** Platelet-related biomarkers of sepsis severity in human studies. MOF, multi-organ failure; ALI, acute lung injury; TPO, thrombopoietin. Modified from [6].

Biomarker	Associated with
Thrombocytopenia	Mortality [7]
Impaired platelet function	MOF [8]
Impaired platelet aggregation	MOF and mortality [9]
sP-selectin	ALI [10]
Platelet-neutrophil aggregates	MOF [11,12]
Immature platelet fraction	Sepsis progression [13]
TPO	MOF [14]

**Table 2:** Ongoing RCTs (randomized controlled trials) involving aspirin in sepsis treatment or prevention.

Trial	Drug	Purpose	Area	Phase	Ref
ANTISEPSIS	ASA 100 mg	Prevention of MOF	Australia	4	[86]
Aspirin for treatment of sepsis	ASA 200 mg	Treatment of MOF	Brazil	2	[87]
LIPS-A	ASA 325 mg then 81 mg	Treatment of ARDS	US	2b–completed	[85]
STAR	ASA 75 mg	Treatment of ARDS	UK	2	[88]

## Abbreviations

ICU	Intensive Care Unit
ARDS	Acute respiratory distress syndrome
AKI	Acute kidney injury
RCT	Randomised controlled trial
DIC	Disseminated intravascular coagulation
TPO	Thrombopoietin
